# Cas13d: A New Molecular Scissor for Transcriptome Engineering

**DOI:** 10.3389/fcell.2022.866800

**Published:** 2022-03-31

**Authors:** Rahul Gupta, Arijit Ghosh, Rudra Chakravarti, Rajveer Singh, Velayutham Ravichandiran, Snehasikta Swarnakar, Dipanjan Ghosh

**Affiliations:** ^1^ Infectious Diseases and Immunology Division, CSIR-Indian Institute of Chemical Biology, Kolkata, India; ^2^ National Institute of Pharmaceutical Education and Research, Kolkata, India

**Keywords:** CRISPR, Cas13d, RNA editing, transcriptome engineering, CasRx

## Abstract

The discovery of Clustered Regularly Interspaced Palindromic Repeats (CRISPR) and its associated Cas endonucleases in bacterial and archaeal species allowed scientists to modify, utilized, and revolutionize this tool for genetic alterations in any species. Especially the type II CRISPR-Cas9 system has been extensively studied and utilized for precise and efficient DNA manipulation in plant and mammalian systems over the past few decades. Further, the discovery of the type V CRISPR-Cas12 (Cpf1) system provides more flexibility and precision in DNA manipulation in prokaryotes, plants, and animals. However, much effort has been made to employ and utilize the above CRISPR tools for RNA manipulation but the ability of Cas9 and Cas12 to cut DNA involves the nuisance of off-target effects on genes and thus may not be employed in all RNA-targeting applications. Therefore, the search for new and diverse Cas effectors which can precisely detect and manipulate the targeted RNA begins and this led to the discovery of a novel RNA targeting class 2, type VI CRISPR-Cas13 system. The CRISPR-Cas13 system consists of single RNA-guided Cas13 effector nucleases that solely target single-stranded RNA (ssRNA) in a programmable way without altering the DNA. The Cas13 effectors family comprises four subtypes (a-d) and each subtype has distinctive primary sequence divergence except the two consensuses Higher eukaryotes and prokaryotes nucleotide-binding domain (HEPN) that includes RNase motifs i.e. R-X4-6-H. These two HEPN domains are solely responsible for executing targetable RNA cleavage activity with high efficiency. Further, recent studies have shown that Cas13d exhibits higher efficiency and specificity in cleaving targeted RNA in the mammalian system compared to other Cas13 endonucleases of the Cas13 enzyme family. In addition to that, Cas13d has shown additional advantages over other Cas13 variants, structurally as well as functionally which makes it a prominent and superlative tool for RNA engineering and editing. Therefore considering the advantages of Cas13d over previously characterized Cas13 subtypes, in this review, we encompass the structural and mechanistic properties of type VI CRISPR-Cas13d systems, an overview of the current reported various applications of Cas13d, and the prospects to improve Cas13d based tools for diagnostic and therapeutic purposes.

## Introduction

### Origin and Advancement of CRISPR

**FIGURE 1:**
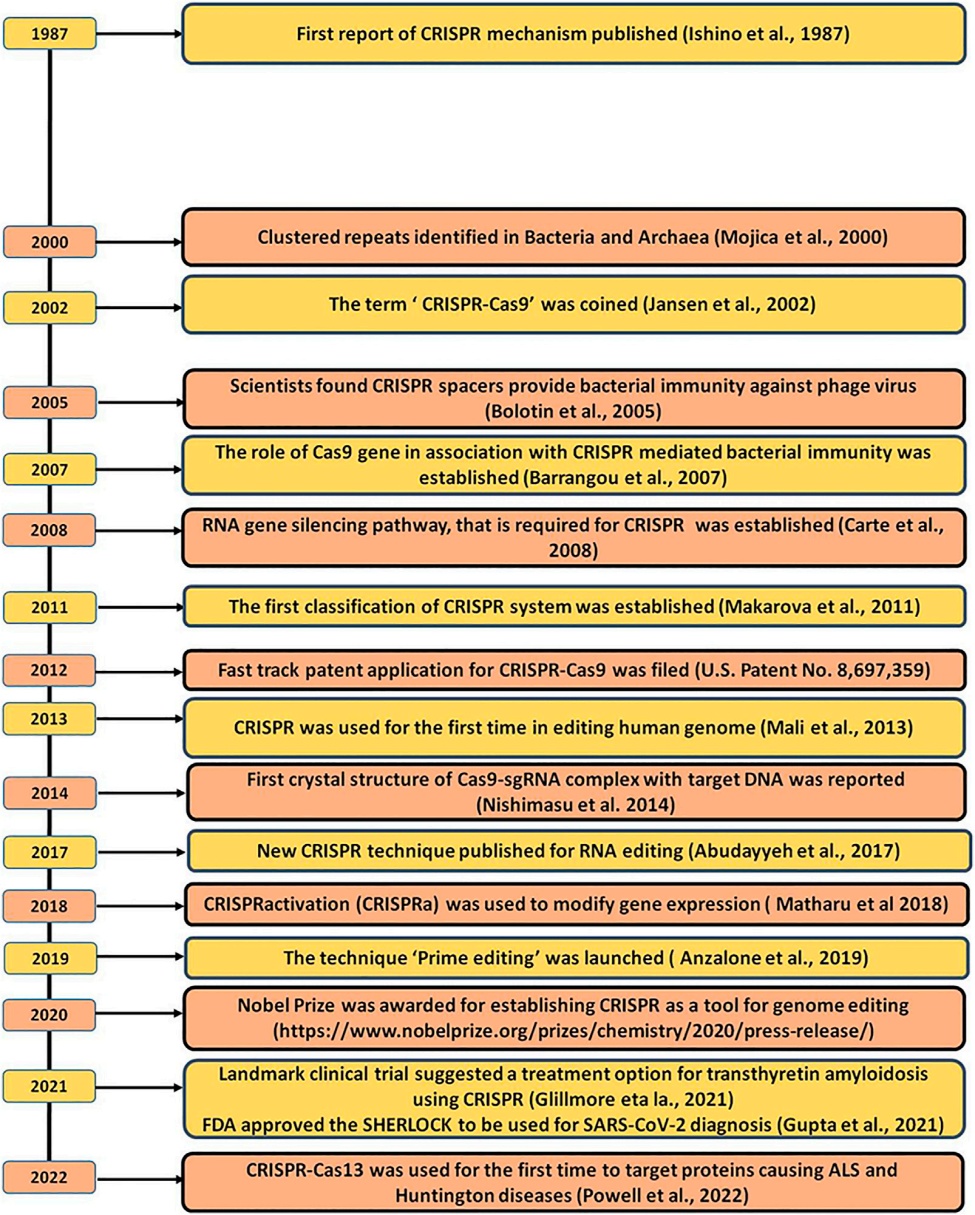
CRISPR timeline, from origin to therapeutics.

CRISPR was first accidentally discovered in *E. coli* back in the year 1987 by the Japanese scientist, Yoshizumi Ishino but due to insufficient DNA sequence data, they were unable to elucidate the function of these repeated arrays (Ishino et al., 2018). In 1993, Francisco Mojica was the first one who characterized and identified 30 bp long DNA sequences that repeat at a regular distance in the genome of halophilic archaea i.e., *Haloferax mediterranei* and called these repeats as short regularly spaced repeats (SRSR) (Mojica et al., 1993). Later, these SRSRs were given the name “CRISPR” by Mojica correspondence with Ruud Jansen, in 2002 (Mojica et al., 2000; Jansen et al., 2002; Mojica et al., 2005; Pourcel et al., 2005; Mojica and Rodriguez-Valera, 2016). Soon after these findings, several CRISPR scientists reported that the Cascade of Cas proteins cleaves the precursor CRISPR RNA (pre-crRNA) to mature CRISPR RNA (crRNA) which then act as a small guide RNAs (gRNA), that guide Cas9 to cleave the viral DNA or any targeted DNA at the 3-nt upstream of protospacer adjacent motif (PAM) sequence, resulting in the precise blunt end, double-stranded breaks (DSB) in the target DNA sequence (Bolotin et al., 2005; Barrangou et al., 2007; Brouns et al., 2008; Garneau et al., 2010). Another pioneer finding in the evolution of CRISPR occurred in 2011, Emmanuelle Charpentier and others discovered a new RNA in the CRISPR-Cas9 system of *Streptococcus pyogenes* called “*trans*-activating CRISPR RNA” (tracrRNA). Once it got transcribed, it interacts with the crRNA and forms a duplex structure that is found to play a critical role in guiding Cas9 to its targeted DNA (Deltcheva et al., 2011) and this discovery finally concluded the CRISPR-Cas9 mechanism to destroy the targeted viral DNA and thus acting as an adaptive immunity for bacterial species. In 2011, the first classification of the CRISPR-Cas system was prepared (Makarova et al., 2011). In 2012, Emmanuelle Charpentier along with Jennifer Doudna engineered a single RNA chimera also called single-guide RNA (sgRNA) by fusing the two RNA i.e., tracrRNA and crRNA which further simplify the system and make it easier to deliver into the host cell. Further, they also concluded that this sgRNA can be designed synthetically and can able to direct the Cas9 to cleave the target double-strand DNA (dsDNA) with high specificity (Gasiunas et al., 2012; Jinek et al., 2012). The main breakthrough came in 2013 when Zhang and his group for the first time showed the ability of the CRISPR-Cas9 system to manipulate the eukaryotic genome loci in mouse and human cells and demonstrated that multiple guide sequences can be incorporated into a single CRISPR array and thus, can be programmed to edit multiple target sites present within the mammalian genome (Cong et al., 2013; Mali et al., 2013). In 2014, two independent groups carried out the knockout of genes that are responsible for cancer viability in mammalian cells and mice models and concluded that CRISPR-Cas9 is a robust tool to perform genome-wide screening with Cas9 (Shalem et al., 2014; Wang et al., 2014). In the same year, Nishimasu and others resolute the crystal structure of Cas9-sgRNA binary complex with target DNA and found two independent nuclease domains i.e., HNH and RuvC domain which is responsible for causing cleavage in the target DNA (Nishimasu et al., 2014). These structural insights helped the scientists over the years to further engineer the Cas9 protein and utilized engineered Cas9 proteins such as Nickase (nCas9) and dead-Cas9 (dCas9) beyond genome editing (Ran et al., 2013; Brocken et al., 2018). Further, the discovery of Cpf1 (Cas12a) in 2015 provides more flexibility to the scientist to choose new target sites which cannot be targeted using Cas9 such as in the malaria parasite and to some extent to humans as well (Zetsche et al., 2015). In 2016, a new CRISPR system discovered type VI CRISPR Cas system which has a novel Cas13 endonuclease that can detect and cleave RNA without altering the genome (Abudayyeh et al., 2016). Finally, in 2020, Jennifer Doudna and Emmanuelle Charpentier won the Nobel Prize for introducing and utilizing the CRISPR Cas9 method to edit DNA^1^. Moreover, recently CRISPR tools have been approved by the FDA to be used in clinical diagnostic as a molecular detector against various viral diseases and even the most recent deadly virus SARS-CoV2 (COVID-19) (Gupta et al., 2021b). Both of these events have given immense hope to the CRISPR scientists that the CRISPR tool will be soon available in clinical practices to treat and control any diseases with ease but still, it is a long way to go. The milestones of the evolution of CRISPR from its origin to its genome editing applications have been briefed in Figure 1.

### General Mechanism of CRISPR Cas: Acquiring, Adapting, Interference

Bacterial and archaeal species utilized CRISPR-Cas systems to defend foreign genetic elements from invading phages and nucleic acids. CRISPR-Cas acts as an adaptive immunity and mainly involves three main stages: 1) *adaptation and spacer acquisition*, where the small size of the invader genome is integrated into the CRISPR array proximal to the 5′ leader sequence; 2) *biogenesis* is the process in which CRISPR array generates pre-CRISPR RNA (pre-crRNA) which is then converted into mature crRNA and thus provides targeting specificity; and 3) *interference*, where the crRNA binds with the effector Cas protein and forms ribonucleocomplex which finds and binds the target invader’s genome with the help of crRNA (guide RNA) and finally causes cleavage or degradation of the invader’s genome (DNA/RNA) (Amitai and Sorek, 2016; Han and She, 2017; Gupta et al., 2019).

### Elementary Classification of CRISPR-Cas System

**FIGURE 2:**
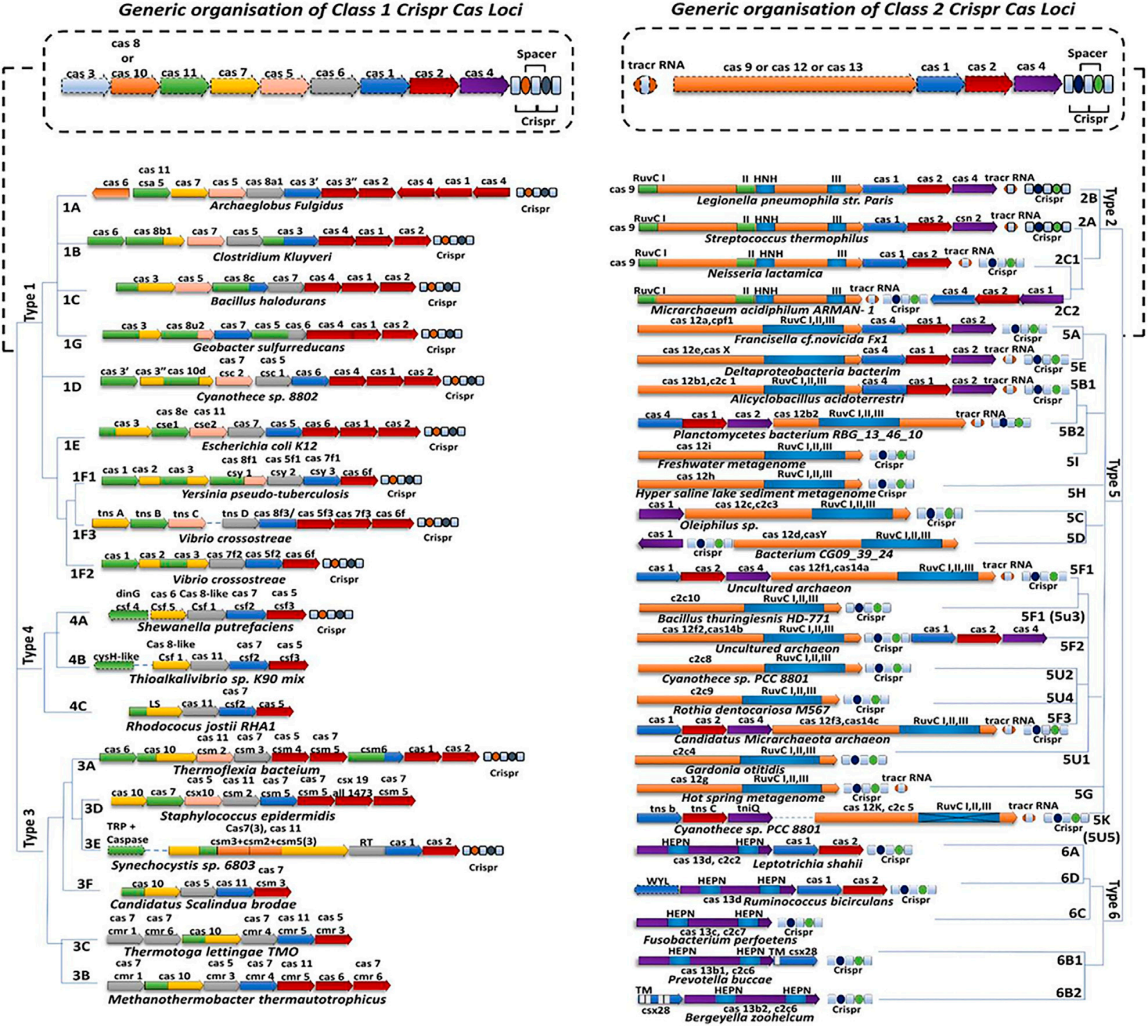
Classification of CRISPR-Cas system. The CRISPR systems can be classified into two broad classes: Class 1 CRISPR system which consists of Type I, III, IV, and their subtypes whereas Class 2 CRISPR system which consists of Type II, V, VI, and their subtypes.

The new classification is based on the phylogeny arrangement and sequence composition of the effector modules and the distinctive Cas genes which are signature endonucleases for specific types and subtypes of the CRISPR-Cas system (Figure 2). These signatures include cas3 for type I, cas10 for type III, cas9 for type II, Csf1 for type IV, cas12 for type V, and cas13 for type VI. Therefore, combining the above two criteria, the CRISPR-Cas system is functionally partitioned into two major classes and each class has three distinct types and each type has several subtypes. Class 1 requires a complex of multi-Cas effector nucleases for nucleic acid cleavage and includes three major CRISPR-Cas system types: type I, III, and IV, and each type has distinctive signature Cas endonucleases: Cas3, Cas10, and csf1 (large subunit, cas8-like) respectively while class 2 requires only one single-Cas effector nuclease for nucleic acid cleavage and consist of three major CRISPR-Cas system types: Type II, V, and VI and each type has distinctive signature Cas endonucleases: Cas9, Cas12, and Cas13 respectively. However, computational pipeline and cryoEM reveal that Cas1 and Cas2 are universal that reside in all CRISPR-Cas system loci (Makarova et al., 2011; Koonin et al., 2017; Makarova et al., 2018). Since class 1 CRISPR system was found to play role in providing adaptive immunity to bacteria and archaea and has also been utilized by the CRISPR scientist for genome editing in several instances but due to the Cas complexity, limited knowledge, the large size of the Cas complex further makes it difficult to clone in functional vector or pack in as a ribonucleoprotein protein (RNP) complex and thus fails to be used as a preferred class of genome editing tool as compared to Class 2 CRISPR system which overcomes the above hurdles.

### The Best-Known Class 2 Types CRISPR-Cas System

Class 2 CRISPR systems are well known and most extensively studied class because of the simpler organization of effector modules, as it includes single-Cas effector protein. This single-Cas effector protein is a multi-domain and multi-functional protein that can be customized and fine-tuned to edit/modify any targeted DNA/RNA substrates with great specificity (Makarova et al., 2006). Hence making it a suitable and potent tool in genetic engineering and allowing scientists to translate the CRISPR-Cas mechanism into use for therapeutic purposes. Now according to the arrangement of the diverse domains of effector proteins and the factors necessary for processing of the pre-crRNA, class 2 is being subclassified into three major types II, V, and VI (Chylinski et al., 2014). Each of these types is discussed and explained below section.

#### Type II CRISPR-Cas System

The type II system is the most abundant and comprehensively studied type in all CRISPR-Cas systems. Further, it has been the first and most broadly used new-generation genome editing tool in the past few decades. The type II CRISPR system consists of a single-effector, multi-domain protein called Cas9 which can recognize the target sequences of the nucleic acid substrates and finally mortify them (Jinek et al., 2012; Esvelt et al., 2013). However, recent studies have revealed that an additional accessory effector and Cas proteins are also required along with signature Cas9 to perform functional nuclease activity and this lead to further classify type II into three subtypes type II-A (csn), type II-B (cas4), type II-C (no accessory protein) (Chylinski et al., 2014). However, out of these subtypes, type II-A [CRISPR-(*Streptococcus pyogenes*) spCas9] is the most useful system for targeted genome editing and is well-characterized, structurally, and functionally (Morange, 2015; Gupta et al., 2019). The components of the *Streptococcus pyogenes* Type II CRISPR system mainly includes CRISPR arrays, RNA-guided spCas9 protein which posses helicase and nuclease activity, two non-coding RNAs–crRNA and tracrRNA which can be engineered and fuse together to generate single-guide RNA (sgRNA) molecule, and the protospacer-adjacent motif (PAM) sequence present at the 3′ end of the targeted DNA sequence (Shah et al., 2013; Doudna and Charpentier, 2014).

##### General Mechanism of CRISPR-Cas9 System

**FIGURE 3:**
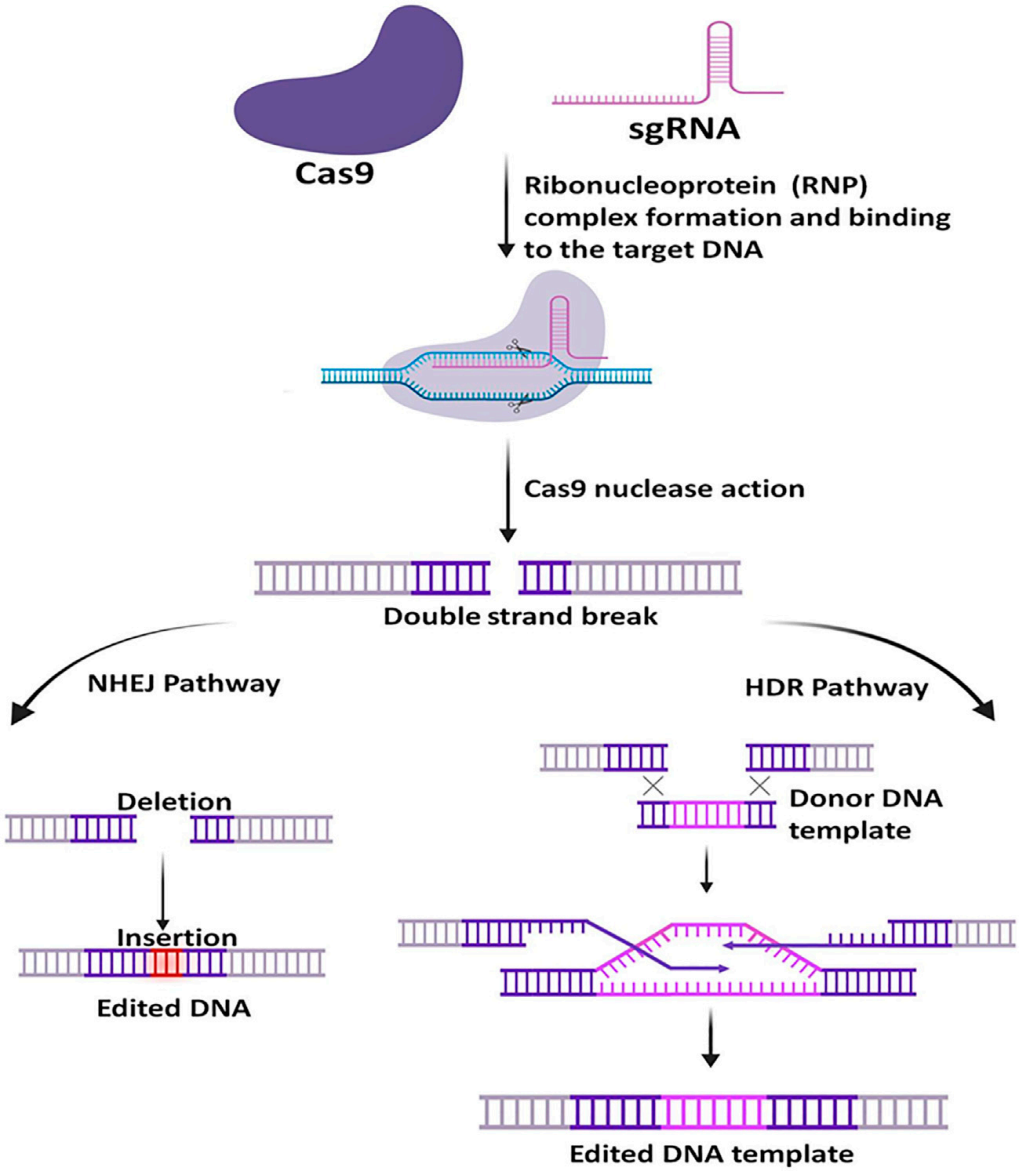
General mechanism of CRISPR-Cas9. At first, the target-specific sgRNA forms complex with Cas9, then the active RNP complex binds with the specific target DNA and creates a double-strand break. Upon Cleavage, the DNA is repaired via two pathways, the non-homologous end-joining (NHEJ) pathway or the homology-directed repair (HDR) pathway. NHEJ is more frequent in nature and is error-prone, whereas the HDR requires a homology template and is less error-prone.

Once the sgRNA binds to the Cas9, it forms an active Cas9 ribonucleoprotein (RNP) complex. This active RNP binary complex stochastically scans the PAM sequence (5′-NGG-3′) on the target DNA sequence and binds to it and forms an active ternary complex, this escorts the activation of two nuclease cleavage domain of the Cas9: the HNH domain and the RuvC-like domain. The HNH domain cleaves the complementary strand and the RuvC domain cleaves the non-complementary strand of the targeted DNA, to the gRNA sequence and thus generating Double-Stranded Break (DSB). The DSB then undergoes repair with two kinds of endogenous DNA repair mechanisms: non-homologous end-joining (NHEJ) pathway or the homology-directed repair (HDR) pathway (Figure 3) (Makarova, 2002; Gasiunas et al., 2012; Jinek et al., 2012; Esvelt et al., 2013; Anders et al., 2014; Doudna and Charpentier, 2014; Nishimasu et al., 2014; Adli, 2018; Gupta et al., 2019; Mosterd and Moineau, 2020). Naturally, NHEJ is the most frequent and efficient repair pathway than the HDR as it is found to remain active throughout the cell cycle. However, studies have shown that the HDR efficiency can be increased by using NHEJ inhibitors to some extent (Gupta et al., 2021a).

##### Utilization of Cas9 Beyond Genome Editing

**FIGURE 4:**
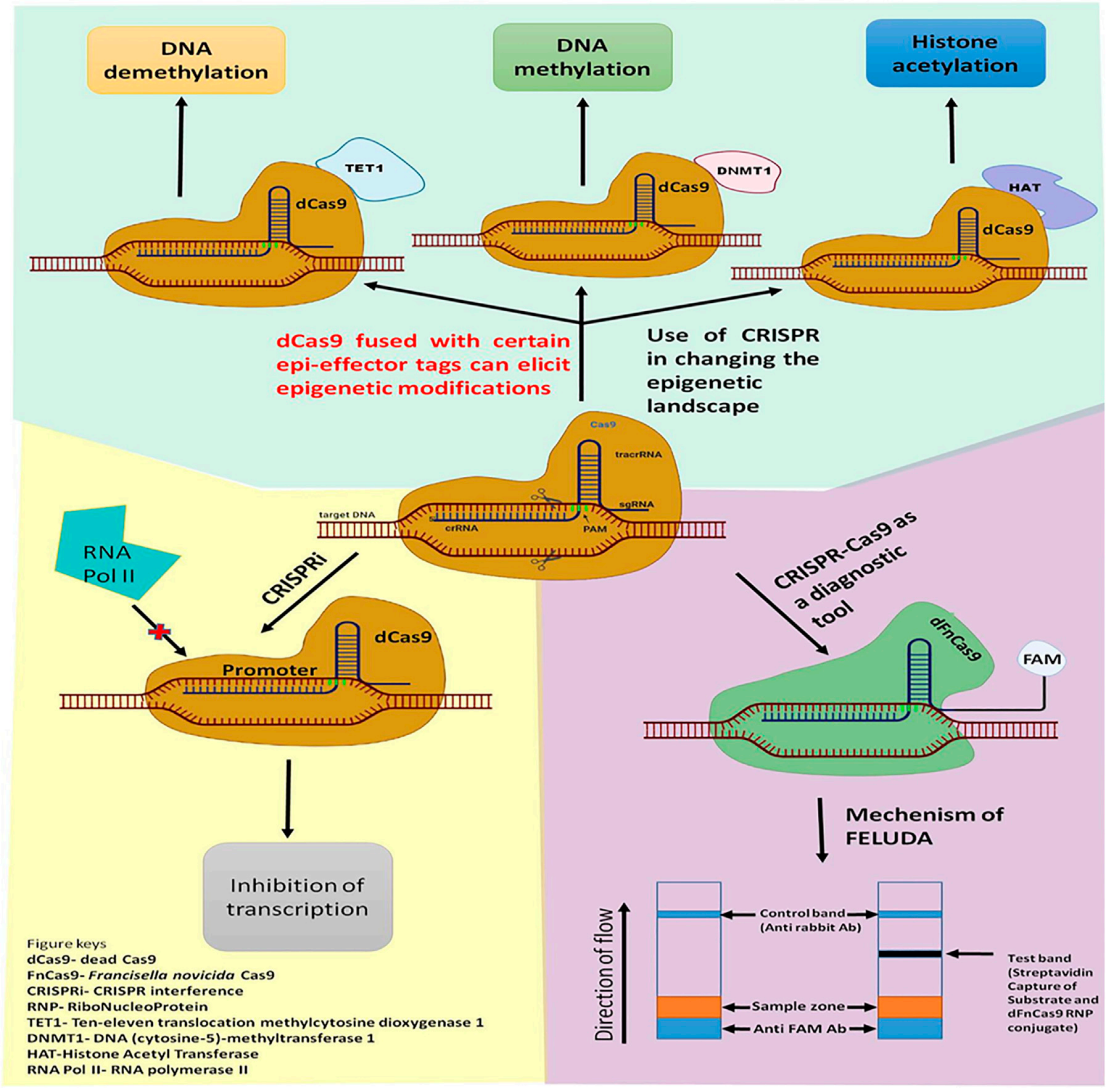
Application of CRISPR-Cas9 beyond genome editing. CRISPR-Cas9 tool can be used for eliciting Epigenetic changes such as DNA methylation or Histone modifications; Gene expression profile can also be modulated using CRISPRi technology. recently, Modified Cas9 like dFnCas9 can be used as a diagnostic tool like FELUDA with high efficacy.

The type II CRISPR-Cas9 system is most widely used and most accepted because it is highly flexible to target and cleave any DNA sequence of the broad spectrum of species, to get an enviable editing outcome with unprecedented ease and specificity (Esvelt et al., 2013; Gilbert et al., 2013; Golkar and Rochelle, 2016). Despite such significant pro features, the CRISPR-Cas9 system still has a severe issue that is currently acting as a barrier in its therapeutic purpose because it smites surplus sites of a similar sequence of DNA other than target DNA, leading to produce off-target effects which may augment genetic flux resulting in an unwanted phenotype (Li et al., 2019). However, a tremendous effort has been made by the CRISPR scientist to overcome these hurdles by adopting the subsequent stratagem: i) Protein engineering/Re-engineering of Cas9 ii) Customization and Modification of guide-RNA (gRNA), and iii) Customization of PAM sequence specificity. Each of these stratagems was found to reduce the off-target effects very efficiently and facilitate precise genome editing (Fu et al., 2014; Kleinstiver et al., 2015; Tycko et al., 2016; Ryan et al., 2017; Gupta et al., 2021a). Further, the crystal structure of Cas9 allows scientists to re-engineer, modify, and utilize it beyond genome editing. These engineered Cas9 proteins are Nickase (nCas9) and dead-Cas9 (dCas9). These nCas9 and dCas9 are generated by inactivating either one or both the nuclease domains (HNH and RuvC) of Cas9 by site-directed mutagenesis. These nickases (nCas9) can be used to generate DSB like wild-type (WT) spCas9 in the targeted DNA sequence if it is used as a paired form. However, instead of blunt ends, it produces long staggered ends which are repaired by the high fidelity base excision repair pathway which is less error-prone and thus, found to reduce the off-target effects by 100 to 1,500 fold as compared to the wild type SpCas9 (Ran et al., 2013). In addition to that nCas9 has been utilized beyond genome editing, as a DNA base editor and also as a gene regulator by fusing nCas9 with base alteration enzyme such as an adenine deaminase, cytidine deaminase, uracil glycosylase inhibitor (UGI), which successfully helps to convert cytidine (C) to thymine (T), adenine (A) to guanine (G), and vice-versa in a targetable manner without altering genome (Hilton et al., 2015; Barrangou and Doudna, 2016). Similarly, dCas9 has been utilized in several applications beyond genome editing such as by fusing it with transcriptional activators or repressors to activate or inhibit targeted gene expression, effector proteins to fuse with fluorescent proteins for genome/chromatin imaging, epigenetic modifiers for regulation of epigenetic modification (Chakravarti et al., 2022), and also can be fused with deaminase to function as base-editor to the targeted sites (Figure 4) (Chen et al., 2014; Gilbert et al., 2014; Brocken et al., 2018; Bharathkumar et al., 2021).

#### Type-V CRISPR-Cas System

In 2013, Class 2 Type V systems were first identified in the human infecting bacterium *Francisella novicida* which has a single signature effector protein, Cpf1 now known as Cas12a, and then it was first characterized by the group of a researcher in Zhang’s Lab at MIT in 2015. Further, researchers have found high diversity in the evolution of Type V system using in silico technique and further sub-classified the Type V system into 10 sub-types from Type V-A to Type V-I and the new series of novel Type V-U (V-U1, V-U2, V-U3, V-U4, and V-U5) system. Each subtype was found to have dissimilar Cas12 proteins because of distinct domain organization and diverge RuvC sequences, distinct targetable substrates, cleavage pattern, distinctive PAM sequence, and composition of gRNA (Zetsche et al., 2015). However, the most studied, well-characterized, and extensively used Type-V system is type V-A (Cas12a). Like cas9, Cas12a protein is a bi-lobed protein that consists of recognition (REC) and nuclease (NUC) lobes. Furthermore, the crystal structure of Cas12a reveals that the REC lobes consist of 2 dsDNA recognition domains, REC1 and REC2 and in the NUC lobe of Cas12a consist of two RuvC domains, a PAM identical (PI) domain, an oligonucleotide-binding (OB) domain, and a NUC [target strand loading (TSL)] domain (Gao et al., 2016).

##### General Mechanism of CRISPR-Cas12a System

**FIGURE 5:**
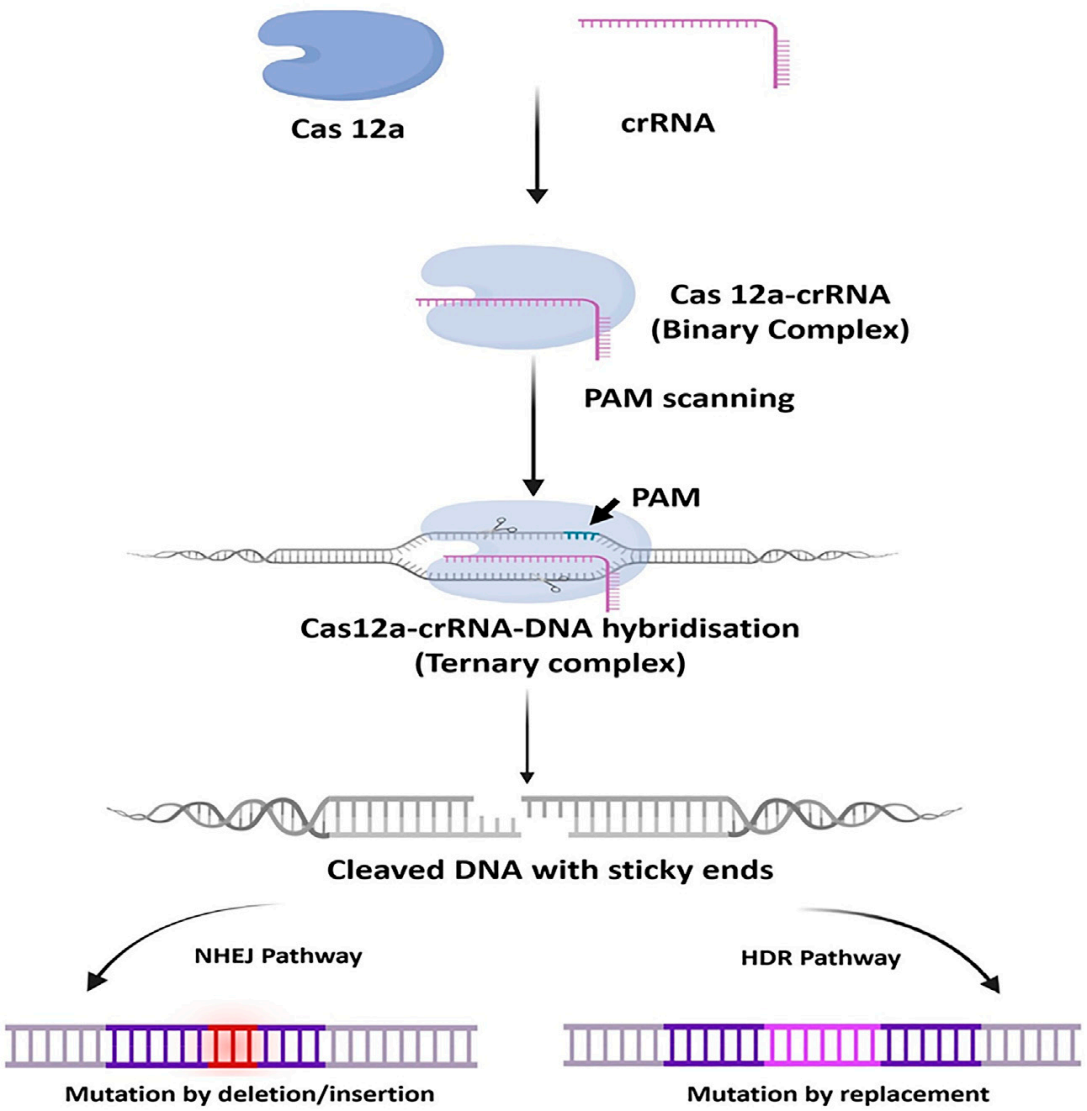
General mechanism of CRISPR-Cas12a. At first, Cas12a interacts and binds at 5′-end of the crRNA (gRNA) with the help of the secondary structure and form a binary complex. The binary complex scans and binds to the T-rich PAM (TTTN/TTN/YTN) sequence in the target dsDNA and starts unwinding it, resulting in the formation of ternary complex and conformational changes in the NUC lobe of Cas12a and thus inducing target dsDNA cleavage activity of RuvC domains. and generates staggered 5, 7, or 10 nt 5′ overhang DSB. This DSB undergoes either the NHEJ repair pathway or the HDR pathway.

The OB domain of Cas12a interacts and binds at 5′-end of the crRNA (gRNA) with the help of the secondary structure and form a binary complex. After the formation of binary complex, the PI domain of Cas12a recognizes the T-rich PAM (TTTN/TTN/YTN) sequence and start unwinding of the targeted dsDNA, which immediately initiate Watson–Crick base pairing between the “seed” sequence of the gRNA and the targeted DNA at the proximal end of the PAM sequence, resulting in the formation of R-Loop structure. The formation of the R-Loop structure causes conformational changes in the NUC lobe and this induces target dsDNA cleavage activity of RuvC domains. However, researchers have found that only one RuvC domain carries out cleavage activity of the target and non-target DNA strands (TS and NTS), not by both RuvC. The RuvC domain incises the NTS and TS sequentially at PAM distant sites and generates staggered 5, 7, or 10 nt 5′ overhang DSB. This DSB undergoes either the NHEJ repair pathway or the HDR pathway (Figure 5) (Gao et al., 2016; Yan et al., 2019).

##### Salient Features of Cas12a

Unlike Cas9, Cas12a has distinct structural and functional features: i) Like cas9, Cas12a protein is a bi-lobed protein that consists of recognition (REC) and nuclease (NUC) lobes. However, the NUC lobe of Cas12a was found to have two RuvC nuclease domains instead of HNH domain, and these RuvC domains are arranged in such a way that they can be superimposed; ii) The Cas12a is unique as it also has RNase activity and thus can solely dapper the pre-crRNA to mature-crRNA; iii) The Cas12a recognizes T-rich PAM sequences such as 5′-TTN-3′/5′-TTTN-3′/5′-YTN-3′ in the target DNA sites; iv) The Cas12a cuts at PAM distant sites i.e., away from the recognition site and generate staggered ends. These unique properties and advantages of Cas12a make the CRISPR-Cas12 system, simpler, more precise, and another valuable asset in the genetic manipulation toolbox. Further, as discussed earlier that each type of V-subtypes has dissimilar Cas12 proteins which showed distinct targetable substrates i.e., it can target double-stranded DNA (dsDNA), single-stranded DNA (ssDNA), and even single-stranded RNA (ssRNA). Because of multi-substrate specificity CRISPR-Cas12 system has been used in a wide range of applications such as genome editing, transcriptional regulation, DNA and RNA base editing, detection of nucleic acid (DNA and RNA), detection of small molecules, and the list is growing (Gupta et al., 2021b; Li et al., 2021). However, the mismatch sensitivity, thermal stability, immunogenicity, and off-target effects are still the area of concern and need more *in vitro* and *in vivo* studies before they can be employed for any bio-medical or therapeutic application. Not only that, to date, only Cas12a, Cas12b, and Cas12e crystal structures have been resolved but the other type V family Cas proteins have not been resolved (Tong et al., 2021). Therefore, analysis of these Cas protein structures is needed to know the functional characteristic so that they can be optimized and employed to be used as a genome-editing tool or beyond genome editing.

#### Type-VI CRISPR-Cas System

The type II and type V CRISPR-Cas System has been extensively utilized to edit DNA but when comes to editing RNA, they are not considered to be a potential candidate due to the off-target effects, less efficient, and still possess the nuclease activity to cleave DNA as well. Moreover, these Cas effectors cannot be exploited competently in all RNA-targeting applications. Further, type II and type V CRISPR-Cas System causes permanent disruption of the target gene which cannot be reversed back once the knockout is induced and additionally, DSB in the genomic DNA increases the risk of off-target effects and toxicity as compared to other gene therapy tools which do not require any alteration in the genomic/chromosomal DNA. Therefore, CRISPR scientists started searching for the CRISPR system which can target RNA and can edit or control the gene expression at the spatiotemporal transcriptomic level without neutering the genome and provide better suppleness and more refined RNA exploitation. Recently, using intensive computational and *in silico* approach scientists discovered the Type VI CRISPR Cas system which has brought a new role to CRISPR to edit and target even RNA with great specificity. Like type II and type V CRISPR systems, type VI CRISPR systems use single signature Cas proteins called C2c2 effectors now known as Cas13a (Abudayyeh et al., 2016). The Cas13’s effector was recently found to be the only known Cas endonuclease that absolutely binds and cleave targeted ssRNA. Like Cas9 and Cas12, Cas13 is also a “bilobed” effector protein which consists of one crRNA “Recognition” lobe (REC) and one “Nuclease” lobe (NUC) but with very different biochemical activities. The cryoEM structural analysis reveals that the REC lobe consists of N-terminal domain (NTD) and a Helical-1 domain that helps in binding and recognizing of crRNA while the NUC lobe consists of two higher eukaryotic and prokaryotic nucleotide-binding domains (HEPN) domains that include Helical-2 domain insertion along with a Linker/Helical-3 domain. Unlike RuvC and HNH domain in Cas9 and only RuvC domain in Cas12 were found to be responsible for deoxyribonuclease cleavage activity but Cas13 lacks homology with any of those nuclease domains and has a pair of HEPN domains which are solely responsible to carry out the ribonuclease cleavage activity (Tang and Fu, 2018; Thai and Floor, 2018; Moon et al., 2019). Further, Cas13 possesses unique dual RNase activities i.e., it can catalyze HEPN-independent processing of a pre-crRNA into a mature crRNA and produces HEPN-dependent RNA cleavage activity. The CRISPR-Cas13’s system is mainly composed of dual components: the programmable RNA-guided RNase Cas13 effector protein, and 64–66-nt crRNA (gRNA) which constitute 24–30-nt spacer region which is complementary to the target site of the ssRNA (Granados-Riveron and Aquino-Jarquin, 2018; Sheridan, 2018; Pei and Lu, 2019). The Cas13 assembled with pre-crRNA array and cleaves within the crRNA direct repeat (DR) and forms mature crRNA in a HEPN independent manner. Upon generation of mature crRNA, Cas13 protein identifies the short hairpin structures in the mature crRNA and forms a mature crRNA-guided RNA-targeting effector complex and does not require any *trans*-activating CRISPR RNA (tracrRNA) unlike Cas9 and Cas12. This Cas13-crRNA complex remains nucleolyticly inactive until it binds to the target ssRNA. Once the Cas13-crRNA complex binds to the targeted ssRNA it forms active ternary RNP complex. This ternary RNP complex undergoes conformational changes which trigger the two HEPN (HEPN1 and HEPN2) domains to come close proximity to each other and generate a single catalytic site where it subsequently cleaves targeted ssRNA bearing a complementary sequence following the protospacer-flanking site (PFS) (O’ Connell, 2019; Perculija et al., 2021). Like Cas9 and Cas12, Cas13 require flanking sequence(s) of protospacers termed as PFS which act as analogous to the Cas9 PAM sequence and therefore required for the RNA targeting cleavage activity but recently it has been discovered not all type VI-Cas13 system requires PFS and this provides more flexibility to target and cleave any RNA sequence with ease. However, in the case of bacteria, once the Cas13 effectors get activated it not only cleaves the targeted ssRNA (*cis* cleavage) but also has the ability to cleave another non-specific ssRNA (*trans* cleavage) regardless of the perfect base-pairing of the crRNA or the presence/absence of a PFS. This non-specific or *trans* cleavage activity is termed as “collateral activity” or “collateral damage” or “collateral cleavage”. On the other hand, such “collateral cleavage” by Cas13 has not been detected in mammalian cells and even in plants, making it a pro and robust tool for spatiotemporal transcriptional editing and manipulation in the eukaryotic system (O’ Connell, 2019; Burmistrz et al., 2020; Kling, 2020; Zaccara et al., 2019; Palaz et al., 2021).

The type VI Cas13 system is further sub-classified into four subtypes VI-A, VI-B, VI-C, and VI-D, based on the presence of adaptation genes (Cas1/Cas2), extra ORF region, associated accessory proteins, and finally due to the significant divergence at the primary sequence except for the conserved two HEPN domains sequence. Each subtype consists of respective Cas13 effectors designated as Cas13a (C2c2), Cas13b, Cas13c, and Cas13d. Moreover, Cas13a, Cas13b, and most recently discovered Cas13d have been well characterized (structurally and functionally) and have been used extensively in various RNA engineering applications. On the other hand, due to the lack of structural and functional information of Cas13c, making it a less comprehensive tool for RNA manipulation as compared to other Cas13 subtypes (Hajizadeh Dastjerdi et al., 2019; Zhang and You, 2020). However, the newly discovered Cas13d has shown several advantages over other Cas13 variants such as its small size which makes Cas13d an ideal and compatible for packaging into a viral vector and carried-out efficient delivery in the mammalian system; neurotoxicity effect of Cas13d founds to be less or absent in mammalian system; Cas13d has dual nuclease activity that is it can cleave targeted ssRNA and can process maturation of crRNA simultaneously; Cas13d does not require protospacer flanking sequence (PFS) for RNA cleavage activity and it does not show off-target transcriptional perturbations as compared to other Cas13 variants. These distinctive features of Cas13d make a superlative choice for spatiotemporal transcriptome engineering, nucleic acid detection, multiplex gene regulation, post-transcriptional gene silencing, alternative splicing, tracking, and RNA epigenetic regulation. Therefore considering the above advantages of CRISPR Cas13d over the previously characterized other Cas 13 subtypes, we in this review encompass the structural and mechanistic properties of type VI CRISPR-Cas13d systems, advantages CRISPR-Cas13d systems over other Cas13 subtypes, an overview of the current reported applications, and the future prospects to improve Cas13d based tools for diagnostic and therapeutic purposes.

## Cas13d and Its Structural Features

**FIGURE 6:**
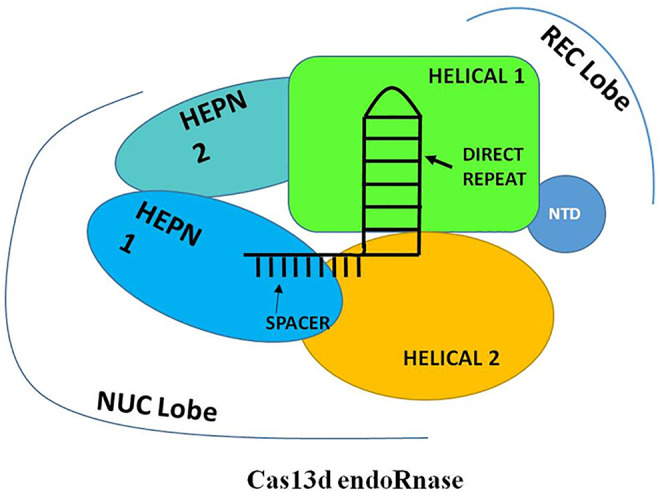
Schematic representation of Generalized structure of Cas13d. The Cas13d is also a bilobed effector protein consisting of one crRNA “Recognition” lobe (REC) and one “Nuclease” lobe (NUC). The REC lobe consists of two domains: the N-terminal domain (NTD) and a Helical-1 domain and the NUC lobe contain 3 domains: two HEPN (HEPN1 and HEPN2) domains and one Helical-2 domain. The REC lobe mainly functions in recognizing and interacting with the stem duplex crRNA repeat and a spacer region of crRNA while the NUC lobe primarily functions in stabilizing the binding of Cas13d-crRNA-target RNA ternary complex and carrying out nucleolytic cleavage of targeted ssRNA.

The application of new improved bioinformatic mining strategies for the CRISPR system and expanding the investigation for smaller effectors led to the discovery of an uncharacterized class 2, type VI CRISPR-Cas system which encodes a novel CRISPR-associated ribonuclease named as “Cas13d” or “type VI-D”. The CRISPR loci of type VI-D is mostly isolated and structurally studied mainly from the two Gram-positive, anaerobic, gut bacteria which belong to the genera of *Eubacterium* and *Ruminococcus*. Recently, the computational and cryoEM studies of Cas13d reveal that it is the smallest endoRNases with ≈930 amino acids in size and bears no significant sequence similarity to other Cas13 effectors except the two conserved R-X_4-6_-H HEPN motif (HEPN1 and HEPN2) domains (Konermann et al., 2018). Further, due to its small size, Cas13d demonstrates a compact architecture as compared to other Cas13 effector molecules. Unlike type VI-A and type VI-B loci, the arrangement of the CRISPR locus of the type VI-D has shown prominent divergence as it lacks spacer adaptation/acquisition effector Cas1–Cas2 protein in their relative vicinity (Zhang et al., 2018). Recent structural studies of Cas13d divulge that like other types VI effectors, Cas13d is also a bilobed effector protein consisting of one crRNA “Recognition” lobe (REC) and one “Nuclease” lobe (NUC). However, the structural arrangement of domains in each lobe was found to resemble slightly like Cas13a but very distantly with Cas13b. The REC lobe of the Cas13d effector consists of two domains: the N-terminal domain (NTD) and a Helical-1 domain. The NTD consists of 7-stranded anti-parallel beta-sheets and two short alpha-helices while the Helical-1 domain consists of 8 alpha-helices. The NTD and the Helical-1 domain mainly function in recognizing and interacting with the stem duplex crRNA repeat and a spacer region of crRNA. On the contrary, the NUC lobe contains 3 domains: two HEPN (HEPN1 and HEPN2) domains and one Helical-2 domain (Zhang B. et al., 2019). The HEPN1 domain is made up of 10 alpha-helices while HEPN2 consists of 11 alpha-helices which further interact with each other through the alpha-3 helix of HEPN1 and alpha-28 helix of HEPN2. The Helical-2 domain is composed of 8 alpha-helices which finally wrap around the alpha-28 helix of the HEPN2 domain. The two HEPN and the Helical-2 domain of the NUC lobe primarily function in stabilizing the binding of Cas13d-crRNA-target RNA ternary complex and carried out nucleolytic cleavage of targeted ssRNA. However, the functioning of all the five domains of Cas13d effectors is found to be vital for its endoRNase activity and only limited truncation is found to be tolerance in the region of the Helical-2 domain. Recent studies also reveal that the HEPN1 domain act as a hinge by providing a structural scaffold to interconnect the 2 lobes of the Cas13d and the HEPN2 domain not only provides a catalytic site for RNA cleavage but also provides a catalytic site for the cleavage of pre-crRNA (Figure 6). Therefore, Cas13d also acts as a dual nuclease effector protein that can able to catalyze pre-crRNA into a mature crRNA and produces RNA cleavage activity like other Cas13 endoRNases. Moreover, the majority of the type VI-D orthologues loci were found to harbor an additional accessory component called the WYL domain. This WYL domain founds to play a crucial role in enhancing and auxiliary modulating the ssRNA cleavage activity in a dose-dependent manner (Yan et al., 2018; Zhang et al., 2018; Chuang et al., 2021).

## Mechanism of Cas13d RNA Cleavage Activity

**FIGURE 7:**
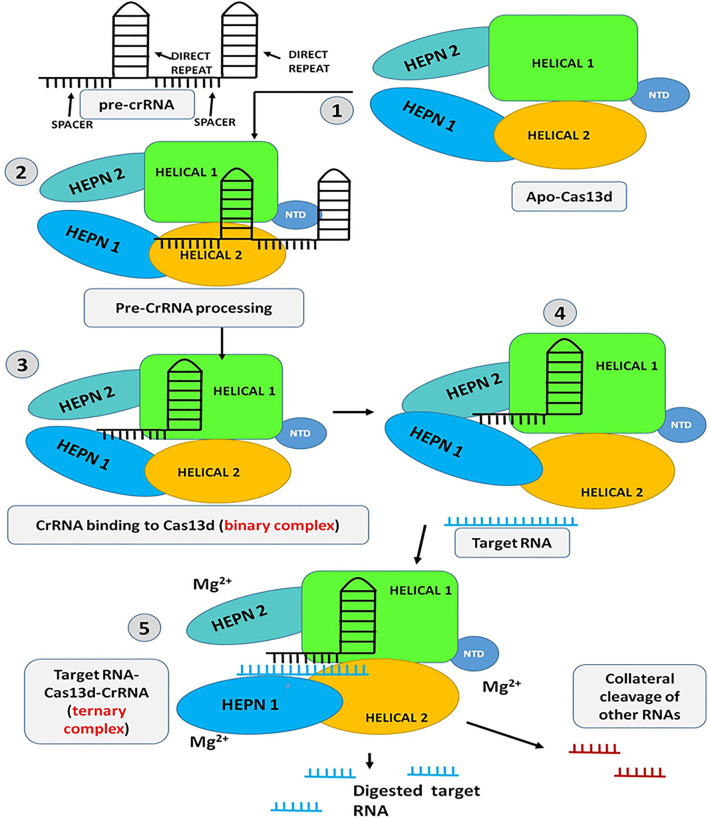
Schematic representation of the mechanism of Cas13d: step 1 to 3-Initially the Pre-CrRNA is processed and the crRNA-Cas13d binary complex is formed; step 4 to 5-The HEPN domains open up and allow the target RNA to get inside the catalytic domain and bind with the gRNA (Target RNA-CrRNA-Cas13d ternary complex). Upon complementary binding of the gRNA with the target RNA, the protein is activated in presence of Mg^2+^ and the target RNA is chopped off. The Activated protein can also chop off other non-target RNA (Collateral cleavage).

The Cas13d assembled with pre-crRNA array and cleaves within the crRNA to form mature crRNA consisting of 30 nucleotides, 5′ direct repeat (DR) region, and 20 nucleotides, 3′ spacer region that is complementary to the target ssRNA. Upon generation of mature crRNA, Cas13d recognizes the 5′ crRNA handle/DR and clamped the first two base pairs (bp) of it with the help of NTD and HEPN2 domain while the 3′ spacer region remain sandwiched between the Helical-1 and Helical-2 domain. The HEPN1 domain act as a hinge by providing a structural scaffold to interconnect the 2 lobes of the Cas13d resulting in the formation of positively charged solvent-exposed RNA density. The binding of crRNA and Cas13d shows extensive molecular interaction between the sugar-phosphate backbone and nucleobases of Cas13d and the crRNA repeat region. These molecular interactions mainly occur in the 3′- end of the crRNA repeat region and are found to be crucial for correct positioning and proper binding of the crRNA with the Cas13d (Konermann et al., 2018; Zhang et al., 2018). Further, both structure and sequence of the crRNA repeat are very much essential and critical for the target ssRNA cleavage activity of Cas13d. Recent studies have shown that Mg^2+^ is another factor that plays a crucial role in enhancing the binding affinity of the Cas13d and crRNA and also stabilizes the conformation of the Cas13d-crRNA repeat region but Mg^2+^ has no effect in the processing of pre-crRNA by Cas13d. The binding of crRNA with the Cas13d resulted in the half-open clam shape solvent-exposed binary complex also called as “surveillance complex”. This Cas13d-crRNA complex (binary complex or surveillance complex) is now ready to actively search and identify the complementary targeted ssRNA (Yan et al., 2018, Hajizadeh Dastjerdi et al., 2019; Zhang and You, 2020). The three U-shaped turns in the 3′-crRNA spacer region and its interaction with the Cas13d helps to further stabilize the conformation of the binary complex and make it available to bind with the targeted ssRNA. This Cas13d-crRNA binary complex remains nucleolyticly inactive until it binds to the target ssRNA. Upon finding the target ssRNA, most of the interaction between the spacer region and the Cas13d gets eradicated and allows the spacer to make a new interaction with the targeted ssRNA to form a double-stranded A-form RNA helix. At the same time, all the domains of the Cas13d further make a new interaction with the sugar-phosphate backbones of crRNA spacer and the targeted ssRNA within the central cleft bound by the HEPN-1, Helical-1, and Helical-2 domains and thus, form Cas13d-crRNA-target ssRNA ternary complex. This ternary complex is also called as “cleavage competent” complex because the HEPN1 and HEPN2 domain with two R-X_4-6_-H motifs forms a catalytic endoRNase HEPN-domain dimer which resulted in the formation of the bipartite active site to arbitrate the hydrolysis of the target as well as collateral ssRNAs. On the contrary, such “collateral cleavage’ by Cas13d has not been detected in mammalian cells and even in plants (Figure 7) (Min et al., 2019; Chuang et al., 2021). Furthermore, studies have shown some distinct requirement to get optimal Cas13d endoRNase activity such as i) HEPN nuclease activity of Cas13d is strongly depend on the base pairing of target ssRNA i.e., binding of *>*21-nucleotide complementarity with crRNA spacer causes full conformational activation and optimal cleavage activity of Cas13d while 18–20 nucleotide complementarity causes half-maximal cleavage activity; ii) presence of any mismatches in the two separate crRNA spacer region (an internal region (spacer nucleotides 5–8) and 3′-end region (spacer nucleotides 13–22) with target ssRNA, founds to be intolerant and completely eradicate the ssRNA cleavage activity; iii) presence of Mg^2+^ and additional accessory component WYL domain increases the endoRNase activity; iv) Cas13d tends to cleave only structurally accessible ssRNA sequences, any secondary structure present in the target RNA sequence it fails to recognize and the RNA cleavage activity is abolished; v) Cas13d has exhibited no preference for PFS imposed ssRNA cleavage but has shown considerable preference for uracil bases in target ssRNA structures. Moreover, the vigorous cleavage activity of Cas13d has been found to remain functional through a broad range of temperatures i.e., 24–41°C and thus making it a potential tool for use in transcriptome engineering in a wide range of hosts (Bharathkumar et al., 2021; Feng et al., 2021).

## Distinctive Features of Cas13a, Cas13b, and Cas13d

**FIGURE 8:**
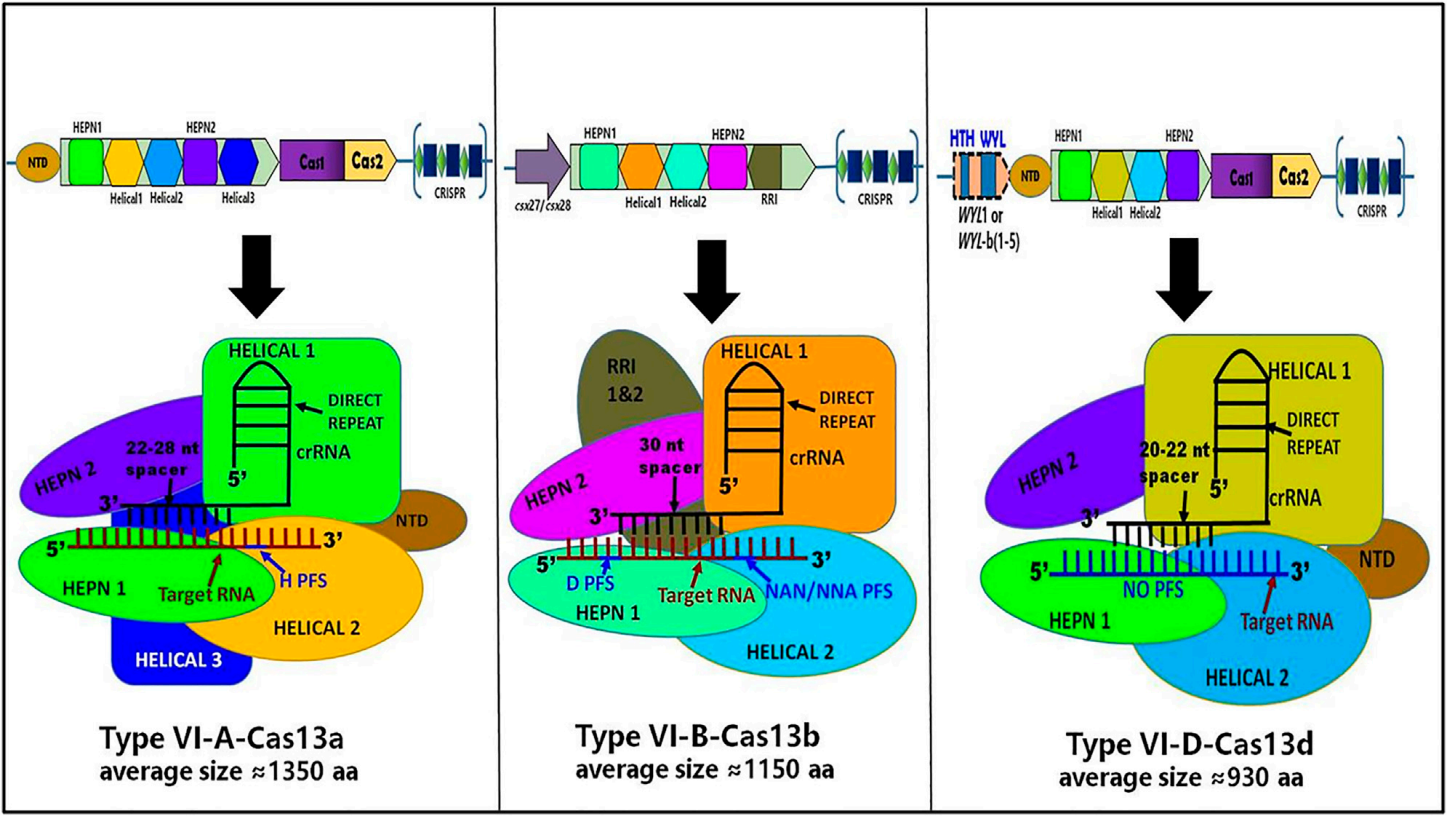
Schematic representation of CRISPR loci and structure of Cas13a, Cas13b, and Cas13d. It shows differences in the genomic and structural organization of respective domains along with their corresponding crRNA spacer size, PFS requirement, and approximated amino acid (aa) size of each Cas13 subtypes: Cas13a, Cas13b, and Cas13d.

Recently, the Cryo-EM structure of apo, binary, and ternary complex of Cas13a, Cas13b, and Cas13d has exposed several structural and functional distinctive features among them, which are discussed below, summarized in Table 1 and shown in Figure 8:

I. The crRNA-target duplex is recognized by two HEPN and two Helical domains in Cas13d whereas in Cas13a, it is recognized inside the pocket formed by HEPN-1, HEPN-2, Helical-2, and Helical-3 domains while in Cas13b may accommodate the crRNA-target duplex within the pocket formed by Helical-1, Helical-2, RRI-1, and HEPN-1 domains.
II. Helical-1 domain of Cas13a is responsible for pre-crRNA processing whereas the RRI-2 domain of Cas13b is responsible for pre-crRNA processing while the counterpart is lacking in the compact Cas13d. Instead, residues from the HEPN-2 domain play a critical role in pre-crRNA processing by Cas13d.
III. In the case of Cas13a, the repeat region of crRNA, which is characterized by stem-loop structure, is recognized by NTD and Helical-1 domains whereas the repeat region of crRNA is recognized by Helical-2, two RRI domains, and the linker region in Cas13b while the repeat region of crRNA is recognized by NTD and two HEPN domains in Cas13d, which still adopts a stem-loop structure.
IV. In Cas13a, a central seed region has been determined whereas the exact seed region of Cas13b still needs to be determined while The seed region may exist in either or both within the internal and the 3′-end spacer regions in Cas13d.
V. There is no requirement of PFS for RNA target cleavage for Cas13d whereas Cas13a requires PFS which includes 5′ non-G while Cas13b requires PFS which includes 5′ non-C or 3′ NAN or NNA
VI. The RNA cleavage activity of Cas13d is found to be modulated by an accessory protein named as WYL domain-containing protein whereas RNA cleavage activity of Cas13d is found to be modulated by an accessory protein, Csx27 and Csx28 while no such accessory protein modulation has been identified in Cas13a RNA cleavage activity.
VII. The size of the Cas13a is approximately ≈1250 amino acids whereas Cas13b is approximately ≈1150 amino acids while Cas13d is approximately ≈930 amino acids and it is the smallest of all Cas13 subtypes.
VIII. The ssRNA cleavage preferences for Cas13d are found to be Uracil (U) whereas ssRNA cleavage preferences for Cas13a are U- and Adenine (A) while ssRNA cleavage preferences for Cas13b are meant to be pyrimidine bases.

**TABLE 1:** Divergence between Cas13a, Cas13b and Cas13d

Properties	Cas13a	Cas13b	Cas13d
Stem length (bp)	5–6	9–14	8–10
Loop length (bp)	7–9	3–6	4–6
Pre-crRNA processing	Helical-1 and Independent	RRI-2 domain and Independent	HEPN-2 domain and Dependent
Recognition of crRNA-target duplex	HEPN-1, HEPN-2, Helical-2, and Helical-3	Helical-1, Helical-2, RRI-1, RRI-2, and HEPN-1 domains	two HEPN and two Helical domains
protospacer-flanking site (PFS) requirement	requires PFS which includes 5′ non-G	requires PFS which includes 5′ non-C or 3′ NAN or NNA	No PFS is required
Cas protein size (amino acid)	≈1250	≈1150	≈930
Presence of modular protein	No accessory proteins were found.	Csx27 and Csx28 accessory proteins	WYL domain-containing proteins

## Advantages of Cas13d Over Other Cas13 Effectors

The newly discovered Cas13d effector has shown several advantages over other Cas13 variants in terms of structure as well as in function: i) Its small size which makes Cas13d an ideal and compatible for packaging into a viral vector and carried-out efficient delivery in the mammalian system; ii) Neurotoxicity effect of Cas13d founds to be less or absent in mammalian system; iii) Cas13d has dual nuclease activity i.e., it can cleave targeted ssRNA and can process maturation of crRNA simultaneously without any requirement of additional helper ribonucleases and tracrRNA; iv) Cas13d does not require protospacer flanking sequence (PFS) for RNA cleavage activity and this makes it lesser sequence constraints and can be used efficiently in targeting of “strongly depleted arrays”; v) Cas13d does not show off-target transcriptional perturbations as compared to other Cas13 variants and exhibits higher efficiency and specificity in cleaving targeted RNA in the mammalian system compared to other Cas13 endonucleases of the Cas13 enzyme family. These superlative features of Cas13d make it a prominent candidate for spatiotemporal transcriptome engineering, nucleic acid detection, multiplex gene regulation, post-transcriptional gene silencing, alternative splicing, tracking, and RNA epigenetic regulation (Yan et al., 2018; Wang et al., 2019; Zhang H. et al., 2019; Kumar et al., 2020; Liu et al., 2020; Qiao et al., 2021; Wu and Kapfhammer, 2021).

## Various Applications of Cas13d in Basic Research and Biomedical Therapeutics

Soon after the discovery of type VI-D, scientists put extensive effort to characterize its biochemical, functional, and structural features because Cas13d (CasRx) is a small novel endoRNase that is highly specific, highly efficient, has autonomous pre-crRNA processing ability, and can be programmed to target any ssRNA sequence with no PFS requirement. This superlative quality of Cas13d makes it a prominent tool for transcriptome engineering, molecular diagnostic, and therapeutic development (Table 2).

**TABLE 2:** Current applications of Cas13d in basic research and biomedical therapeutics.

Study summary	Organism used	Efficiency	Gene involved	References
CRISPR-RfxCas13d system has been implemented on several model organisms to study gene function and developmental changes in different organisms. This study demonstrated that CRISPR-RfxCas13d can be utilized as an efficient knockdown tool to investigate the maternal and zygotic gene function in various animal embryos.	Zebrafish	76%	tbxta, szrd1, dnd1, smad5, alk8, oep, smad2, NANOG, brd3a, brd3b, and brd4	Kushawah et al. (2020)
	Medaka		rx3	
	Killifish		firstly, mRNAs encoding gfp, rfp, mCas13d were introduced, three gRNAs targeting rfp were introduced as well.	
	Mouse		ubtf and emg1	
To develop a programmable platform for RNA targeting of known phenotypic genes using genetically encoded programmable RNA-targeting RfxCas13d (CasRx) system have shown limitation like unexpected toxicity and lethality of endogenously in *Drosophila melanogaster*	Drosophila	NK	Ubiq, NOTCH gene, White gene, Y (Yellow) gene	Buchman et al. (2020)
CRISPR-CasRx system was incorporated in glial cells, *in vivo* and targeted the single RNA-binding protein, polypyrimidine tract-binding protein 1 for the conversion of glia to neuron using CasRx -mediated knockdown of Ptbp1 shows a potential therapeutic approach for tackling neurodegenerative diseases and brain damage due to neuronal loss.	Mouse	76–87%	Ptbp1	Zhou et al. (2020)
dRfxCas13d was incorporated into the neuronal model of frontotemporal dementia cells to maneuver pathological alternative splicing of tau pre-mRNA which result in successful alleviation of dysregulated tau isoform.	Patient-derived human-induced pluripotent stem cells (hiPSCs)	>90%	MAPT	Konermann et al. (2018)
Cas13d nuclease activity was able to efficiently knock-down various exogenous and endogenous genes which are involved in apoptosis, gene amplification, metabolism, and glycosylation.	Chinese hamster ovary (CHO) cell	80–90%	GS, BAK, BAX, PDK1, and FUT8	Shen et al. (2020)
Targeting metabolic genes in mouse hepatocytes by active RfxCas13d, clearly presented to be an efficient strategy to carry-out regulatory knockdown of metabolic genes that can be efficiently used in the treatment of metabolic diseases.	mouse	16–61.6%	Pten, Pcsk9, and lncLstr	He et al. (2020)
CasRx is able to knock down the transcript of mutant KrasG12D and further abolishes the irregular activation of downstream signaling pathways resulting in suppression of tumor growth.	AsPC-1, PANC-1, MIAPaCa-2, and H6c7 Pancreatic cancer cell line and nude mice.	50%	Mutant KrasG12D	Jiang et al. (2020)
Silencing of lncRNA MIR497HG via CRISPR/Cas13d induced bladder cancer progression through promoting the crosstalk between Hippo/Yap and TGF-β/Smad signaling	Human bladder cancer cell lines T24, 5637, RT4, UM-UC-3, SW780, and TCCSUP.	NK	MIR497HG	Zhuang et al. (2020)
Aptazyme mediated CRISPR/Cas13d gene-editing system can efficiently sense hTERT and selectively inhibits the progression of bladder cancer cells	bladder cancer cell line 5,637 and T24	NK	hTERT	Zhuang et al. (2021)
CasRx system successfully constructed an inducible expression system and applied it for efficiently repressing the expression of a green fluorescent protein (GFP) in *E. coli*	*E. coli*	∼70% at mRNA level and 30–50% at the protein level	green fluorescent protein (GFP)	Zhang et al. (2020)
A new tool, the CRISPR-based RNA-United Interacting System (CRUIS) was developed, which captures RNA–protein interactions in living cells by combining the power of CRISPR while fused to proximity enzyme PafA. CRUIS was able to show a similar interactome profile of NORAD (Noncoding RNA activated by DNA damage) and CLIP (crosslinking and immunoprecipitation) based methods.	HEK239T cell line	20–70%	CXCR4, p21, NORAD	Zhang et al. (2020)
CRISPR-Cas13 systems were used to deliver APEX2 to the human telomerase RNA hTR with high specificity enabling RNA interactome profiling on a 1-min time scale. ALKBH5 is able to erase the m^6^A modification on endogenous hTR.		NK	hTR, APEX2, ALKBH5	Han et al. (2020)
Xie et al., found that REMOVER (*reengineered m* ^*1*^ *A modification valid eraser*), a Cas13d based CRISPR tool which specifically demethylated the m^1^A of MALAT1 and PRUNE1 RNAs and also noted that it significantly increased their stability. Thus, their studies have established that REMOVER can be used as a tool for targeted RNA demethylation of specific m^1^A-modified transcripts.	HEK239T cell line	NK	MALAT1 and PRUNE1	Xie et al. (2021)
RfxCas13d coupled with fluorescent-labeled crRNA along with dCas9-fluorescent crRNA system has been used for real-time simultaneous visualization of transcript RNA and genomic DNA in the method known as CRISPR LiveFISH (live-cell fluorescent *in situ* hybridization)	Human osteosarcoma cell line U2OS cell line	NK	PPP1R2, SPACA7	Wang et al. (2019)
RfxCas13d did not exert collateral cleavage effect in plants and was able to efficiently target two RNA viruses in parallel when crRNAs targeting two viruses were expressed in tested plants. CasRx showed robust interference in both transient and stable overexpression assays when compared to the other Cas13 variants tested.	*Nicotiana benthamiana*	NK	Replicase gene of tobacco mosaic virus (TMV) and tobacco rattle virus (TRV)	Mahas et al. (2019)
RfxCas13d (CasRx) in combination with HIV-specific gRNAs efficiently inhibited HIV-1 replication in cell line models.	HEK293T derived Lenti-X^TM^ cells and TZM-bl cell line	>90%	gRNAs having active site of the HIV protease enzyme (BR23), the central polypurine tract (BR29), catalytic core domain of integrase (BR34), and the c-terminal domain (BR04)	Nguyen et al. (2021)
The RfxCas13d based PAC-MAN (prophylactic antiviral CRISPR in human cells) was shown to efficiently cleave SARS-CoV-2 RNA fragments and inhibit the influenza A virus.	A549 and MDCK cells	NK	RdRP or N gene regions of SARS CoV 2 and eight negative-sense RNA segments of IAV including RNA polymerase subunits, hemagglutinin, Nucleoprotein, Neuraminidase, M1, M2, NS1, and NEP	Abbott et al. (2020)
Two Cas13d orthologs were introduced for detection of low variant allele fraction, 0.1% T790M. Overall, this study demonstrated that both EsCas13d and RspCas13d could robustly detect target RNA carrying special single-nucleotide variation with high specificity and sensitivity.	*E. coli* BL21 (DE3)	72%	EGFR	Qiao et al. (2021)

NK-signifies that the efficiency of knockdown or targeting efficiency of Cas13d is not known.

### Exploitation Cas13d in Developmental Studies

Recently, a Type VI-D CRISPR-Cas system has been implemented on several model organisms to study gene function and developmental changes in an organism using Cas13d knock-down and also determine cytotoxic and off-target effects, of Cas13d. Kushawah et al., evaluated the gene function in the early development of teleost embryos using type VI CRISPR-Cas systems and found that among all the Cas13 effectors, RfxCas13d (CasRx) efficiently and precisely degrade the specific mRNA transcript of a maternal and zygotic gene in zebrafish embryos. The efficiency of knock-down was observed to be 76% without producing any cytotoxicity and any abnormalities in development. Further, they also noted that there were no collateral cleavage activity and no direct off-target effects. Therefore, their study demonstrated that CRISPR-RfxCas13d can be utilized as an efficient knockdown tool to investigate the maternal and zygotic gene function in various animal embryos (Kushawah et al., 2020). Similarly, Buchman et al., used RfxCas13d (CasRx) endogenously in the model organism *Drosophila melanogaster* and develop a programmable platform for RNA targeting of known phenotypic genes in addition to unexpected toxicity and lethality. They genetically encoded CasRx in flies and they found a moderate decrease in the targeted transcript. They also reported the cellular toxicity, off-target and on-target effects on the transcript cleavage by CasRx and they found that it cannot able to produce homozygous strains due to tissue necrosis and unexpected lethality arrived because of collateral cleavage of CasRx. Overall they have shown the limitation of using genetically encoded programmable RNA-targeting Cas13d system in *Drosophila melanogaster* and thus needs more extensive research and future optimization (Buchman et al., 2020).

### Exploitation Cas13d in Neuronal Diseases

Several neurodegenerative diseases and brain damage were found to be associated with mainly neuronal loss. The current therapeutic approach is to replenish such neuronal loss to convert glial cells into functional neurons and generate desired neuronal types. Zhou et al., incorporated the RNA-targeting CRISPR-CasRx system in glial cells, *in vivo* and targeted the single RNA-binding protein, polypyrimidine tract-binding protein 1 (Ptbp1). They successfully knockdown the Ptbp1, which resulted in the conversion of Muller glia into retinal ganglion cells (RGCs) and thus reducing the disease symptoms associated with RGC loss. They also reported a reduction in a motor defect in a Parkinson’s disease mouse model by inducing neurons with dopaminergic features in the striatum, using a CasRx-mediated approach with high efficiency. Therefore, conversion of glia to neuron using CasRx -mediated knockdown of Ptbp1 shows a potential therapeutic approach for tackling neurodegenerative diseases and brain damage due to neuronal loss (Zhou et al., 2020). Further, Type VI-D CRISPR-Cas systems have also been put into practice to manipulate alternative splicing. Konermann et al., incorporated dRfxCas13d into the AAV vector and transfer it to the neuronal model of frontotemporal dementia cells to maneuver pathological alternative splicing of tau pre-mRNA and they found that dRfxCas13d binds to cis-elements of pre-mRNA of tau and successfully manipulated the alternative splicing which result in alleviation of dysregulated tau isoform ratios in a neuronal model of frontotemporal dementia (Konermann et al., 2018).

### Exploitation of Cas13d Editing in Metabolic Engineering

Researchers have shown that the typeVI-D CRISPR-Cas system has significantly been used in metabolic and cell engineering in order to treat a metabolic disorder by studying metabolic gene function and also to increase biopharmaceutical production. Recently, Shen et al., utilize the CRISPR-Cas13d system for gene silencing, attenuating glycosylation, and Chinese hamster ovary (CHO) cell engineering of Chinese hamster ovary (CHO) cells. Their study demonstrated that Cas13d nuclease efficiently knock-down various exogenous and endogenous genes which are involved in apoptosis, gene amplification, metabolism, and glycosylation such as GS, BAK, BAX, PDK1, and FUT8 in CHO cells. They design specific gRNA for GS, BAK, BAX, PDK1, and FUT8 and co-transfected with Cas13d in CHO cells and they observed that the efficiency of knock-down of these genes was about 80–90% and was able to generate stable engineered CHO cells. Further, knockdown of the FUT8 gene causes attenuation of IgG fucosylation with 90% efficiency. Thus, their study has shown the generation of stable engineered CHO cells using the CRISPR-Cas13d platform, which has the ability to attenuate lactate accumulation and glycosylation and also shown apoptosis resistance which resulted in an increase in cell titer, enhance recombinant protein and antibody production during fed-batch culture (Shen et al., 2020). Similarly, He et al., used active RfxCas13d to target metabolic genes including Pten, Pcsk9, and lncLstr, in mouse hepatocytes. They delivered RfxCas13d and sgRNA of each metabolic gene into the mouse liver via AAV vector for simultaneous and reversible knockdown of RNA transcripts which resulted in a significant reduction of serum cholesterol levels and this is due to the decrease of PCSK9 in the serum. Therefore, their study clearly presented the efficient strategy to carry-out regulatory knockdown of target metabolic genes and laid the foundation for type VI-D CRISPR-Cas systems to be efficiently used in the treatment of metabolic diseases (He et al., 2020).

### Exploitation of Cas13d Editing in Cancer

As the structural and functional advancement in typeVI-D is in rapid progression, type VI-D is successfully being employed in several cancers related research. (Jiang et al., 2020). applied the CRISPR-CasRx system to knock down the mutant KrasG12D transcript in Pancreatic ductal adenocarcinoma (PDAC) cells because more than 90% of PDAC cancers were found to have a Kras mutation. Out of several mutations in Kras, the single-site mutation G12D (KrasG12D) is the most ubiquitous one. They incorporated the CRISPR-CasRx system and specific KrasG12D-gRNA into a capsid-optimized adenovirus-associated virus 8 vectors (AAV8) and deliver the AAV8 vector into PDAC orthotopic tumors and patient-derived tumor xenografts (PDX). They found that the CasRx is able to knock down the transcript of mutant KrasG12D and further abolishes the irregular activation of downstream signaling pathway and subsequently resulting in suppression of tumor growth. Further, the silencing of the mutant KrasG12D expression also improves the sensitivity of gemcitabine in PDAC cells and increases the survival rate of mice without any observable toxicity. Additionally, delivering CasRx-gRNA via AAV8 into the orthotopic KrasG12D PDAC tumors substantially improves the survival of mice without obvious toxicity. Therefore, CRISPR-CasRx can be exploited for programmable knock-down of any mutant transcripts and consequently inhibit tumorigenesis (Jiang et al., 2020). Similarly, Zhuang et al., evaluated the effect of lnc-miRHGs MIR497HG and its molecular mechanisms in bladder cancer (BCa). First, they used the in silico approach to finding the expression of two harbored miRNAs (miR-497 and miR-195) and lnc-miRHG MIR497HG is downregulated in BCa cells and then they co-transfected the BCa cells with Cas13d and the specific MIR497HG-gRNA to find the effect of knockdown of MIR497HG. They reported that the knockdown of MIR497HG in BCa cells using Cas13d significantly increases cell growth, migration, and invasion *in vitro.* Further, they noticed that miR-497 and miR-195 jointly suppress the Hippo/Yap pathway by reducing the interaction between Yap and Smad3 and finally affecting the binding of the E2F4 which plays a critical role in silencing MIR497HG transcription in BCa cells. Their studies showed a potential strategy for the therapeutic treatment of BCa by knocking down of lnc-miRHGs MIR497HG using the CRISPR/Cas13d system (Zhuang et al., 2020). Recently, Zhuang et al., employed the concept of Aptazyme and CRISPR/Cas gene-editing system and constructed an engineered CRISPR/Cas13d tool that can efficiently sense hTERT and selectively inhibits the progression of bladder cancer cells. The method also employs the use of OFF-switch hTERT aptazyme incorporated into the 3′ UTR of the Cas13d, which can further inhibit the degradation of Cas13d in bladder cancer cells. Further, their study also revealed that the incorporation of reconstructed CRISPR/Cas13d sensing hTERT in bladder cancer 5637 and T24 cells was found to induce cell apoptosis and subsequently inhibit cell proliferation, migration, and invasion without affecting normal human foreskin fibroblast (HFF) cells (Zhuang et al., 2021). Hence, CRISPR/Cas13d sensing hTERT approach can serve as a prominent therapy to control the tumorogenesis of bladder cancer.

### Exploitation of Cas13d Editing in Bacteria

Due to the high activity of Cas13d (CasRx), Zhang et al., have recently employed the usage of the CRISPR-Cas13d system to carry out the knockdown of RNA in bacterial cells. They constructed an inducible expression system for CasRx with a tightly controlled promoter and a weakened ribosome binding site and incorporated it into *Escherichia coli* (*E. coli*) to target green fluorescent protein (GFP) RNA. They further optimize the approach by optimizing the inducer usage and manipulating guide RNA (gRNA) structural design to achieve the highest knockdown efficiency. However, they found that the highest knockdown efficiency of GFP at the mRNA level was ∼70% and at the protein level was 30–50% and considered it to be moderate repression. Moreover, this moderate repression of RNA is not only achieved by targeted RNA cleavage but also by the collateral cleavage activity toward bystander RNAs and thus torment bacterial metabolism (Zhang et al., 2020).

### Exploitation of Cas13d in Post-Transcriptional Modification, RNA Visualization, and RNA–Protein Interactions Studies

The inactivation of the nuclease activity of HEPN domains of Cas13d leads to the generation of catalytically dead-Cas13d (dCas13d) which can recognize and bind to the target ssRNA but lacks cleavage activity. This dcas13d has further allowed the CRISPR researchers to utilize the CRISPR-Cas13d system beyond the RNA editing. Recently, Cas13d has been employed for the mapping of RNA–protein interactions *in vitro*. Recently, Han et al., utilizes specific crRNA along with dead-RfxCas13d (dRfxCas13d), fused with modified plant peroxidase APEX2 (ascorbate peroxidase) with the help of the dsRNA-binding domain to target selected RNA, resulting in biotinylated labeling of transitorily interacting proteins and these interacting partners were analyzed using liquid chromatography-coupled tandem-mass spectrometry (Han et al., 2020). Similarly, Yi et al., discovered a new method called CAPRID (CRISPR–CasRx-based RNA targeting and proximity labeling) which uses a similar approach and founds to identify proteins binding to specific nuclear lncRNAs in mouse cells with high specificity. Both of these approaches were successful in identifying and mapping endogenous RNA–protein interactions and further showed advantages over other RNA–protein interactions methods because they do not require any cross-linker or any genomic manipulation of the targeted RNA (Yi et al., 2020).

The adenosine methylation/demethylation found to play a vital role in regulating the fate of RNA and gene expression but due to the lack of methods and tools to produce site-specific adenosine methylation in specific mRNA, found to be the roadblock to discovering the association between site-specific adenosine methylated/demethylated modified mRNA and its phenotypic outcomes. To fill this technical inadequacy, CRISPR scientists have successfully employed the Cas13d-based tools to understand the involvement of site-specific adenosine methylation/demethylation mediated epitranscriptome regulation in cellular processes and diseases with ease. The most prevalent, abundant, and reversible post-transcriptional mRNA modifications are the N^1^-methyladenosine (m^1^A) and N^6^-methyladenosine (m^6^A) which are founds to regulate several cellular processes such as gene expression, alternate splicing, translation, and degradation of transcripts. Recently, Xie et al., developed a new CRISPR-Cas13d-based tool called “REMOVER” (*reengineered m* ^*1*^ *A modification valid eraser*) to target specific transcripts for m^1^A demethylation. They introduced catalytically inactive RfxCas13d (dCasRx) and fused it with the m^1^A demethylase ALKBH3, and they found that ALKBH3 fused dCasRx protein can mediate specific demethylation of m^1^A of MALAT1 and PRUNE1 RNAs and also noted that it considerably increased their stability. Thus, their studies have established that REMOVER can be used as a tool for targeted RNA demethylation of specific m^1^A-modified transcripts. (Xie et al., 2021).

Likewise, Wang et al. have developed cas13d based tool called CRISPR LiveFISH (live-cell fluorescent *in situ* hybridization). In this method, they used catalytically inactive RfxCas13d (dRfxCas13d) fused with fluorescent-labeled crRNA along with the dCas9-fluorescent crRNA system for monitoring real-time simultaneous visualization of transcript RNA and genomic DNA in the live cell (Wang et al., 2019). The development of such tools makes it a powerful tool to visualize and track any nascent RNAs in the live cells much easier than other visualization and tracking methods. However, this method requires further experimental optimization to accurate the signal-to-noise ratio to make it a more prominent tool for visualization and tracking any RNAs.

### Exploitation of Cas13d as an Antiviral System

The discovery of Cas13d endoRnase has further extended the applications to detect and fight against the emerging deadly viruses by targeting and cleaving the viral RNAs with the specific crRNA in virally infected cells in mammalian as well as in plant systems with high specificity and thus acting as an antiviral agent. Mahas et al., transiently and stably express *Leptotrichia wadei* (LwaCas13a), *Prevotella* sp. (PspCas13b), and *Ruminococcus flavefaciens* (RfxCas13d) in Nicotiana benthamiana and target both the plant RNA viruses [tobacco rattle virus (TRV) and tobacco mosaic virus (TMV)] with appropriate crRNAs respectively. They found in transient assays LwaCas13a, PspCas13b, and CasRx variants arbitrate high interference activities against both the RNA viruses but CasRx founds to mediate more robust RNA interference activities against RNA viruses in both transient and stable overexpression assays as compared to other cas13 variants. Further, CasRx does not found to produce any collateral cleavage activity in planta and thus demonstrates strong specificity against the targeted virus and thus they conclude that CasRx is the most prominent endoRNase as compared to other Cas13 variants for RNA virus interference applications in planta (Mahas et al., 2019). Similarly, Abbott et al., has developed a new antiviral tool called PAC-MAN (prophylactic antiviral CRISPR in human cells) which is based on RfxCas13d isolated from *Ruminococcus flavefaciens*. They used PAC-MAN to target multiple highly conserved genomic regions of SARS-CoV-2 and influenza A virus, as well as the majority of other coronavirus and influenza virus strains, and found that the RfxCas13d efficiently cleaves the targeted viral RNAs and inhibits viral multiplication in human lung epithelial cells, even with the lower viral titers. As a result, PACMAN could be used efficiently to combat new viral infections or can be used as an alternative genetic vaccine approach to destroy the intracellular viral genome and its mRNAs and thus faltering the viral replication in humans without the need to inject the virus itself (Abbott et al., 2020). Moreover, recently Nguyen et al., evaluated the effectiveness of CRISPR/Cas13d to stall the replication of the HIV-1 virus. Primarily, they incorporated and expressed the RfxCas13d (CasRx) along with the highly specific guide RNAs (gRNAs) that target the highly conserved regions of HIV-1 in HIV-1 infected primary CD4^+^ T-cells and the cell line models. Further, they reported that RfxCas13d (CasRx) significantly and efficiently inhibited the HIV-1 replication when targeting four separate, non-overlapping sites in the HIV-1 transcript in all the cell line models as well in primary CD4^+^ T-cells. Therefore their study concluded that the use of the CRISPR/Cas13d nuclease system to target acute and latent HIV infection will endow with an unconventional treatment modality against HIV (Nguyen et al., 2021).

### Exploitation of Cas13d as a Molecular Diagnostic Tool

All Cas13 variants exhibit targeted endoRNase activity, but once it gets activated after recognition and binding to the target ssRNA, it remains activated and exhibit collateral activity, leading to non-specific degradation of any nearby transcripts despite complementarity to the spacer and this property of Cas13s has been harnessed to detect nucleic acid (ssRNA) in 30–45 min with high specificity and accuracy. Recently, Qiao et al., used two Cas13d effectors from *Eubacterium siraeum* (Es) and *Ruminococcus* sp. (Rsp) and applied them to detect nucleic acid and they found that both the Cas13d exhibit efficient and rapid target RNA detection in picomolar range and even in the lower viral titer. On the whole, their study revealed that both EsCas13d and RspCas13d were able to detect the targeted RNA and even it can detect single-nucleotide variation with high sensitivity and specificity (Qiao et al., 2021). Therefore, the CRISPR-Cas13d system can be utilized as a newly qualified molecular diagnostic tool to detect various pathogenic viruses and can also be used to identify biomarker genes for various diseases.

These experimental results represented that Cas13d (CasRx) can be used as a programmable RNA-binding module for efficient targeting of cellular RNA, enabling a general platform for transcriptome engineering and future diagnostic and therapeutic development.

## The Current Known Transcriptome Engineering Tools

RNA editing is a post-transcriptional process that is broadly defined as any site-targeted alteration due to processes such as insertion and deletion of nucleotides, conversion of one base to another, splicing, etc. RNA-targeted genetic manipulation is capable of controlling target-protein functions, similar to genome editing, without the risk of damaging the original genomic information. Molecular tools to manipulate and measure RNA are limited. Though Small-interfering RNAs (siRNAs) and microRNAs (miRNA) can knock-down the RNA expression but its off-target effect is very much a matter of concern. In contrast to RNA-interference technology, a general RNA-mutagenesis technology enabling RNA modification is in its developing stage. In cellular machinery there are some enzymes, like two Adenosine Deaminases Acting on RNA (ADAR): ADAR1 and ADAR2, which convert adenosine to inosine (A → I) (Gott and Emeson, 2000; Bass, 2002). Inosine is recognized as Guanosine by the splicing and translation apparatuses and, thus, ADARs modify amino acid sequences (Patterson and Samuel, 1995; Gott and Emeson, 2000; Desterro et al., 2003; Hogg et al., 2011; Nishikura, 2015). To achieve targeted RNA editing, the ADAR protein—or its deaminase domain ADAR_DD_—has been fused to a variety of RNA-targeting modules, such as AD-gRNA, λN-peptide, SNAP-tag, cas13, etc.

In 2017, Fukuda et al., designed ADAR-guiding RNA (AD-gRNA), comprising a 19 nucleotides-long anti-sense recruiting region (ARR) and a 49 nucleotides-long ADAR-recruiting region (APR), which directly induces A-to-I mutations by docking hADAR2 to the target-site (Fukuda et al., 2017). This was a breakthrough toward the establishment of practical site-directed RNA mutagenesis using gRNA for ADARs. In the same year, the Zhang Feng group also developed programmable editing of RNA transcript to alter their coding potential using a new system named “RNA Editing for Programmable A to I Replacement” (REPAIR) which has no strict sequence constraints and is thus very much effective for editing full-length RNA transcript (Cox et al., 2017). This system engineered catalytically inactive Cas13b (dcas13b), which retained programmable RNA binding activity to direct A to I change using ADAR2. Further modifying the system, they introduced REPAIRv2 which has dramatically higher specificity than previous RNA editing platforms. Later the same group showed that the class 2 type VI6,7 RNA-guided RNA-targeting CRISPR-Cas effector Cas13a from *Leptotrichia wadei* (LwaCas13a) is capable of providing comparable levels of knockdown as RNAi, but with dramatically improved specificity (Abudayyeh et al., 2017; Saifullah et al., 2020).

The above-mentioned targeted RNA editing tools, which rely on ectopic expression of exogenous editing enzymes, the delivery of exogenous proteins, or chemically modified guide RNAs, have many limitations including substantial global off-target editing of the genome and/or RNA transcripts, immunogenicity, oncogenicity, and delivery hurdles. To mitigate the problem Mali group has engineered ADAR-recruiting guide RNAs (cadRNAs) to enable more efficient programmable adenosine-to-inosine RNA editing which recruited endogenous ADARs, and thus, vastly improved the durability of RNA editing. Two more RNA editing technologies, RESTORE (recruiting endogenous ADAR to specific transcripts for oligonucleotide-mediated RNA editing) (Merkle et al., 2019) and LEAPER (leveraging endogenous ADAR for programmable editing of RNA) (Qu et al., 2019), employed endogenous ADARs for programmable editing of RNA. RESTORE is an engineered chemically optimized antisense oligonucleotide that recruits endogenous human ADARs to edit endogenous transcripts in a simple and programmable way without perturbing natural editing homeostasis and off-target effect. Whereas, LEAPER, reported by the Wei group, employs short-engineered ADAR-recruiting RNAs (arRNAs) to recruit native ADAR1 or ADAR2 enzymes to change specific adenosine to inosine and thus enables precise, efficient RNA editing with broad applicability for therapy and basic research. The same group has further improved the LEAPER to an updated LEAPER 2.0 version that uses covalently closed circular arRNAs, termed circ-arRNAs, which enhanced the editing efficiency almost three times more than their linear counterparts (Yi et al., 2022).

## Future Perspective and Conclusion

It is undeniable that CRISPR will be an essential tool for genome and epigenome editing in the near future. Even though Cas9 stole all the limelight initially, it has limited therapeutic applications owing to its off-target effects. Further, the emergence of cas12 and Cas13 family nucleases, allowed the scientists to utilize CRISPR in several applications such as epigenetic modification, base editor, chromatin imaging, optogenetics, vesicle tracking, gene regulation, and the list is growing (Gupta et al., 2021b). Not only that the CRISPR Cas system has also been used in various biomedical applications to treat diseases such as HIV, Cancers, β-Thalassemia, sickle cell disease (SCD), cystic fibrosis, spinal muscular atrophy (SMA), Leber Congenital Amaurosis (LCA10), Hereditary transthyretin amyloidosis (hATTR), urinary tract infections (UTIs), etc., which are in preclinical and clinical trials (Kotagama et al., 2019; Xu and Li 2020). While the initial endeavors in implementing CRISPR as a genome-editing tool were limited to the editing of DNA, it was seen that this approach had far more deleterious effects on the overall genetic construct. Since the focus shifted towards targeting RNA it was becoming clear that this approach had the upper hand over DNA editing. Unlike DNA editing, RNA editing effects were less prominent and the changes are temporary. It must be noted that although Cas13 based therapies are paving the path for CRISPR as a theranostic tool, the technology is still in its infancy. The system is not perfect, it can induce collateral cleavage and its effective delivery is still challenging. It is still not clear the rampant effect of this collateral cleavage, since this is lacking in eukaryotic organisms. Mechanistically speaking, we have limited understanding of some of the aspects of Cas13d function such as activation of the HEPN domain with respect to target search and also on which active sites of HPEN1 and HEPN2 domains are involved in RNA catalysis; The pre-crRNA processing site of Cas13d has not been identified yet; Due to limited resolutions of the available cryo-EM structures of Cas13d has limited resolution and therefore the higher-resolution structure is needed to know the complete features of Cas13d; Whether the recognization of the repeat region of crRNA by Cas13d is a structure-specific or a sequence-specific manner or both, needs detailed investigation; The mismatch tolerance between the crRNA spacer region and the target RNA needs to be experimentally tested thoroughly; At the molecular level, how the accessory proteins such as WYL1 modulate Cas13d activity is still limited and unknown; Further, delivery of Cas13d components is another area of concern for *in vivo* and human applications but recently some efforts have been made to increase the efficiency of the delivery which include methods such as Cas-inspired artificial CIRTS and nebulization; Moreover, immunogenicity, cytotoxicity, and stability of Cas13d components in the body fluids are the areas that still need to be addressed. Therefore, a better understanding of these processes can help scientists to develop engineered Cas13d nucleases with better efficacy and specificity. It is anticipated that Cas13d will be employed in various avenues but not limited to transcriptome editing. It will be more advanced than traditional CRISPRi approaches in terms of gene regulation. Finally, dCas13d tagged with fluorescence tags can help us visualize allele-specific RNA modifications like splicing, translation, polyadenylation, and degradation. To summarize, Cas13d is a “new molecular scissor” that demands more attention to fully unfold its potential and needs an ingenious solution to overcome the associated challenges to become a gold standard in transcriptome editing and application beyond RNA editing with salutary results in the coming future.

## Footnotes

1 Nobel Prizes 2021. (2020, October 7). [Press release]. https://www.nobelprize.org/prizes/chemistry/2020/press-release/.
